# Behavior Change Pathways to Voluntary Medical Male Circumcision: Narrative Interviews with Circumcision Clients in Zambia

**DOI:** 10.1371/journal.pone.0111602

**Published:** 2014-11-06

**Authors:** Jessica E. Price, Lyson Phiri, Drosin Mulenga, Paul C. Hewett, Stephanie M. Topp, Nicholas Shiliya, Karin Hatzold

**Affiliations:** 1 Population Council, Zambia Office, Lusaka, Zambia; 2 Population Council, HIV and PGY Divisions, New York, New York, United States of America; 3 Centre for Infectious Diseases Research, Lusaka, Zambia; 4 University of Alabama, Health Care Organization & Policy Unit, Birmingham, Alabama, United States of America; 5 University of Melbourne, Nossol Institute for Global Health, Melbourne, VIC, Australia; 6 Population Services International, Research, Monitoring and Evaluation, Lusaka, Zambia; 7 Population Services International, HIV and SRH, Harare, Zimbabwe; International AIDS Vaccine Initiative, United States of America

## Abstract

As an HIV prevention strategy, the scale-up of voluntary medical male circumcision (VMMC) is underway in 14 countries in Africa. For prevention impact, these countries must perform millions of circumcisions in adolescent and adult men before 2015. Although acceptability of VMMC in the region is well documented and service delivery efforts have proven successful, countries remain behind in meeting circumcision targets. A better understanding of men's VMMC-seeking behaviors and experiences is needed to improve communication and interventions to accelerate uptake. To this end, we conducted semi-structured interviews with 40 clients waiting for surgical circumcision at clinics in Zambia. Based on Stages of Change behavioral theory, men were asked to recount how they learned about adult circumcision, why they decided it was right for them, what they feared most, how they overcame their fears, and the steps they took to make it to the clinic that day. Thematic analysis across all cases allowed us to identify key behavior change triggers while within-case analysis elucidated variants of one predominant behavior change pattern. Major stages included: awareness and critical belief adjustment, norming pressures and personalization of advantages, a period of fear management and finally VMMC-seeking. Qualitative comparative analysis of ever-married and never-married men revealed important similarities and differences between the two groups. Unprompted, 17 of the men described one to four failed prior attempts to become circumcised. Experienced more frequently by older men, failed VMMC attempts were often due to service-side barriers. Findings highlight intervention opportunities to increase VMMC uptake. Reaching uncircumcised men via close male friends and female sex partners and tailoring messages to stage-specific concerns and needs would help accelerate men's movement through the behavior change process. Expanding service access is also needed to meet current demand. Improving clinic efficiencies and introducing time-saving procedures and advance scheduling options should be considered.

## Introduction

Multiple studies, including three randomized control trials [1]–[3], have demonstrated the efficacy of voluntary medical male circumcision (VMMC) in preventing HIV acquisition in heterosexual men. Lowered risk of viral and bacterial sexually transmitted infections in circumcised men is also recognized and the indirect heath benefit passed on to female partners is considered to be substantial [4]. In countries with high HIV burden and low circumcision levels, VMMC has been recommended as a key HIV prevention strategy and service delivery scale-up is now underway in 14 priority countries in east and southern Africa [5]. To maximize the prevention benefit of VMMC, these countries will need to perform over 20 million circumcisions by 2015 [4].

Previous research has indicated high reported rates of acceptability of male circumcision for prevention of HIV [6]–[8]. In a review of the acceptability studies in nine countries in Eastern and Southern Africa, Westercamp and Bailey [8] found a median percentage of uncircumcised males willing to be circumcised at 65%, although support varied from a low of 29% in Uganda to 87% in Swaziland. The key barriers were determined to be pain, culture or religion, cost and fear of complications, among others. In Zambia, where approximately 13% of the population was circumcised prior the study, mostly traditionally [9], Lukobo and Bailey [7] found widespread recognition of the relationship between male circumcision and HIV prevention and further report a general willingness among men to become circumcised or to have their sons circumcised. Although some men in Zambia expressed that circumcision is limited to the two traditionally circumcising tribes, the authors indicate that respondents were capable of making the distinction between traditional circumcision during adolescence and medical circumcision for HIV prevention purposes.

Despite reports of high acceptability of VMMC in the priority country populations most countries are lagging behind in meeting their targets [10]. Global health leaders have called for further research that will help explain the low uptake of VMMC compared to coverage targets [4]. The present study responds to this call.

While it is generally assumed that low VMMC uptake requires more demand generation [11], early service delivery experiences [12]–[14] indicate a need to nuance this assumption. Matching the supply of VMMC services to fluctuating demand levels, offering services in places and at times that are convenient for men, and addressing disproportionate uptake between younger (less at-risk) and older (more at-risk) men are issues highlighted in case studies on service delivery roll-out [12]–[14]. Beyond these observations made in early service delivery efforts, little else is known about VMMC demand and the psycho-social factors that lead men to seek the procedure.

While a variety of studies have been conducted on the acceptability of male circumcision, few have focused specifically on VMMC demand and uptake in the context of a scaling program. Based on published findings from VMMC acceptability studies, one secondary analysis of VMMC uptake inventoried a wide range of individual- and community- (socio-cultural) level barriers and facilitators [15]. Based on their review the authors propose several interventions to increase VMMC uptake. Another secondary analysis was conducted by Gummerson and colleagues [16]. Interested in learning about the socio-demographic characteristics of VMMC adopters, these researchers compared two rounds of Demographic and Health Survey data from Tanzania and confirmed that VMMC rates had increased in targeted districts. Their findings also showed that uptake was greatest among younger, more educated, wealthier and less at-risk men. Also in Tanzania, in order to understand facilitators and barriers to VMMC uptake among older men, Plotkin et al. [17] elicited the perceptions of various community members through focus group discussions. Concerns about pain, loss of income in the post-surgical period, and the social unacceptability of attending the same clinics with adolescents were key issues raised by the discussants. Although these studies provide useful insight on VMMC demand, given their respective focus on acceptability [15], on socio-demographic correlates of uptake [16], and on general community perceptions [17], none shed light on how VMMC demand emerges, gets acted on, and gets met or not by the medical sector.

This paper, which is based on data from Zambia, adds important clarification on these matters. From an experience-rich sample [18] of adult clients waiting for surgical circumcision in two clinics in Lusaka, we specifically sought to understand VMMC demand and uptake as phenomena that happen as a process. Rather than eliciting static beliefs, perceptions, barriers and facilitators, our phenomenological enquiry focused on the changes that men go through leading them ultimately to VMMC clinics. To this end, we adapted the Transtheoretical Model (TTM) of behavior change [19] to structure narrative interviews with men, in which we asked them to recount their VMMC-seeking trajectories from time they first became aware of adult circumcision to the point of seeking out the procedure for themselves. Centered on the notion that people change their behavior over time, TTM is a flexible theory that has been used to structure a wide variety of health interventions and research [20]–[24]. In our study, by asking men to describe what *actually occurred in their lives over time* we begin to understand VMMC demand and uptake as a series of dynamic personal events that together constitute a process of change. From the collection of behavior change events described by the men in our sample we are able to identify intervention opportunities specific to adult men's changing needs over time.

## Methods

### Sample

In November 2012, 40 men waiting to receive surgical VMMC were interviewed at two outpatient clinics in Lusaka. Men ≥18 years were included in the study. Assuming that marital status would influence the VMMC-seeking process as well as reflect important age differences, we purposively selected for married and unmarried men, aiming for equal representation. To be clear, important experiential and age differences between the two groups of men were anticipated and were the very basis for this purposive selection criterion [18], [25]. While recruitment of unmarried men was completed easily and quickly, lower clinic attendance by married men required us to extend the number of interview days to enroll as close to 20 married men as possible. No other purposive selection criterion was applied.

The clinics were selected because of the relatively high volume of circumcisions they perform, approximately 30 per day. Operated by the same non-profit organization, the clinics are similarly staffed and organized and both specialize in HIV and reproductive health services. Clinical officer and nursing staff perform VMMC surgery during normal operating hours on Mondays through Fridays and during a shortened work-day on Saturdays. Field supervisors coordinated with clinic management and staff to identify and enroll eligible VMMC clients into the study and in a way that did not interrupt service provision. After registering at reception and paying the VMMC fee, clients were approached by a research staff member and asked to participate in the study.

### Interviews and Interviewers

Interviews were conducted in a private space at the clinics prior to the men being called for the surgery and after obtaining their informed consent to participate in the study. Interviews were digitally recorded, each taking between 30 and 40 minutes. Audio recordings were backed up at the end of each day and later uploaded to NVivo version 10.

Open-ended questions were structured around four theorized behavior change stages: (i) exposure and first impressions, (ii) early contemplation, (iii) serious contemplation and early action, and (iv) commitment to becoming circumcised. Men were asked to recount how they learned about VMMC, how and why they decided it was right for them, what they feared most about becoming circumcised, how they overcame their fears, and the steps they took to make it to the clinic that day. The research team translated the guide from English to Nyanja, the predominant local language spoken in Lusaka. Both English and Nyanja versions were pretested and modified as needed (Materials S1 shows the final guide used). Interviewers were equipped with guides in both languages and they conducted the interview in the respondent's preferred language.

All of the interviewers were men, had prior experience working on a male circumcision study, were competent in qualitative interviewing, and were fluent in English and Nyanja. Interviewer training reviewed public health and clinical issues related to male circumcision; covered study objectives, methods and field procedures; and reinforced qualitative interviewing techniques. The interviewers were fully engaged in translating, pretesting and modifying the guide.

Interviews were completed in November 2012.

### Analysis

Data analysis was directed at empirically specifying our theorized behavior change process. Combining analyses from within individual cases and across the whole dataset [26], [27], we carried out the data analysis process in four main steps.

#### Within-case data immersion and *in vivo* coding

Our first analytic step focused on understanding the behavior change trajectories for each of the 40 men. DM, LP and the interviewer jointly listened to each audio-recorded interview and, based on consensus, assigned interview segments to the four theorized behavior change stages using NVivo's audio-coding function. This broad ordering of audio data facilitated transcription and follow-on coding of text data. DM and LP then jointly identified significant behavior change statements in the text data. Significant statements reflected thoughts, situations, events, and relationships that shaped the men's understanding of circumcision and that facilitated or obstructed their movement from one behavior change stage to another. Captured as *in vivo* codes, significant statements were preserved as actual (translated) terms and phrases used by the participants.

#### Across-case thematic analysis

*In vivo* codes were subsequently used to generate successively inclusive thematic categories across all 40 cases. We consolidated like utterances (e.g., *Jesus was circumcised* and *It's not just for Muslims*) into common themes (e.g., *religious belief alignment*). To ensure coding consistency, we again opted for a collaborative approach with DM and LP together deciding on *in vivo* code assignment to common themes. Successive data consolidation resulted in 55 thematic codes, which were subjected to inter-coder reliability checks and collaborative coding. DM and LP independently analyzed five interviews each to determine the presence or absence of each of the 55 themes (Table S1 lists the themes and provides sample quotes). They then independently coded each other's five interviews and, in cases of discrepant coding, came to definitional consensus. The remaining 30 interviews were collaboratively coded using the refined code definitions.

The 55 themes were examined to understand key triggers in the behavior change process: (i) multiple exposures to VMMC messages, (ii) adjustments in beliefs about circumcision and the procedure, (iii) social pressure to become circumcised, (iv) personalization of the advantages of being circumcised, and (v) management of fears about becoming circumcised. These five behavior change triggers structure much of the content of this paper.

Establishing the presence or absence of themes and, in turn, key behavior change triggers for each individual resulted in a “quantitized” case-by-theme matrix, with 1 indicating the presence and 0 indicating the absence of themes cited by each man [28]. This quantitized dataset allowed us to discern the predominant behavior change pattern observed in the study population.

#### Within-case identification of variant behavior change patterns

We returned to within-case analysis to validate the predominant behavioral pattern specified through thematic analysis as well as to identify variations on this pattern. This involved assessing for each man the presence or absence of each of the five key behavior change triggers to determine all variations within the whole population. To complete our understanding of behavior change through to VMMC uptake, we also recorded the total number of prior attempts to obtain VMMC surgery that each man mentioned in his interview.

#### Qualitative comparative analysis between ever- and never-married men

The final analytic step was to ascertain points of commonality and divergence between our purposively selected categories of men: married and unmarried [25]. To more accurately reflect differences in social positions and age, rather than comparing currently married and single (including divorced and widowed) men, we compared **ever**-married and **never**-married men.

#### Ongoing iterative thematic analyses and data verification

In addition to the initial 55 themes discovered through systematic identification of significant behavior change events, additional themes of interest emerged iteratively as our interpretation of the data progressed. We thus frequently returned to the data to carry out additional coding and thematic analysis as needed. To verify translations and to clarify men's descriptions, digital recordings were also frequently reviewed in whole or in part.

### Ethics

Permission to conduct this study was obtained from the University of Zambia's Biomedical Research Ethics Committee and from the Population Council's Institutional Review Board. Following a consenting procedure approved by the Ethics Committee, participants provided written informed consent to participate in the study. Signed consent forms were kept in a secure file accessible only by the research team.

## Results

Table 1 summarizes age and education of the 40 men by marital status. All of the men identified as Christian. Ethnic backgrounds varied, with Bemba being the largest ethnic group (n = 10) followed by Tonga (n = 7), Chewa (n = 7) and various other tribes (n = 16).

**Table 1:** Marital Status, Age, and Educational Background.

	Ever-married (n = 17)	Never-married (n = 23)	Overall (n = 40)
Age (in years)			
Range	23–48	18–29	18–48
Median	33	22	26
Formal education (in years)			
Range	7–18	7–16	7–18
Median	12	13	12

### Overview of the VMMC Seeking Process

With individual variation in context and detail, we observed one predominant behavior change pattern in the data. During a **pre-intention** stage of awareness men learn about adult circumcision and its benefits and adjust their beliefs about the safety and appropriateness of VMMC for themselves. Resulting from both external and internal factors, pre-intention stage awareness shifts to **intention** – a decision – to become circumcised. In this stage of change, men experience substantial social pressure to be circumcised while they also personalize the advantages of VMMC to fit their individual circumstances and perceived needs. But a decision to become circumcised does not produce immediate action. Prior to seeking VMMC surgery men go through variably long processes to confront and conquer their fears about getting the procedure. In time, with VMMC benefits personalized and fears overcome, men take **action** and present at VMMC clinics ready to be circumcised. Showing up at a clinic mentally prepared to go through with the surgery, however, does not necessarily conclude the behavior change process with a completed circumcision. Service-side issues often obstruct men's ability to get the surgery, requiring many to repeat the VMMC-seeking step multiple times.

**Figure 1:**
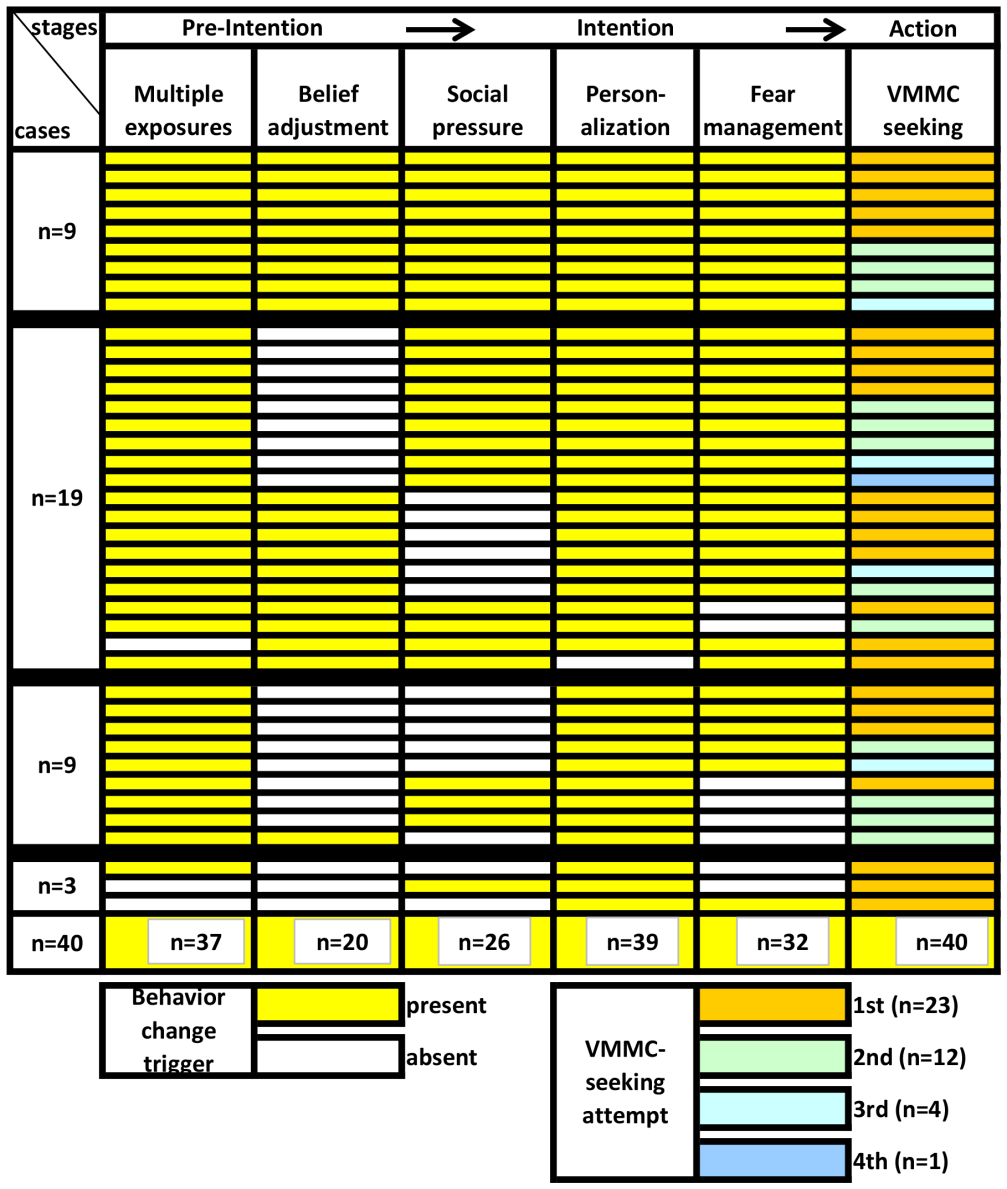
VMMC Behavior Change Triggers and Uptake Within and Across Cases (n = 40).

To validate this overall interpretation of the data we examined conformity to and divergence from the predominant behavior change pattern for each of the 40 men. Figure 1 visualizes this case-by-case examination. Varied experiences at the VMMC-seeking stage are indicated in the last column of the figure which shows the number of attempts each man has made to receive surgical circumcision. Rather than to suggest a linear process of change from one discrete stage to the next, we offer this synthesis to approximate and “see,” in one view, the variable behavior change and VMMC-seeking experiences within our whole study population.

Although the duration of the change process was often not evident in the interviews, it was clear that this varied greatly and could be quite long. Because of his precision and confidence in relating key dates, the example of one 26 year old, never-married man illustrates. The man first heard about VMMC in August 2010 from co-workers and by February 2011 he was convinced that it was right for him. The fear of a long healing period, however, prevented him from acting on his decision for another 21 months. When interviewed for this study, he expressed exasperation at his own self-induced VMMC-seeking delays: *Now it's November 2012 and I feel like I've wasted a lot of time*.

It is from the details of this and other men's stories that we are able to identify communication and intervention opportunities to help men avoid “wasting a lot of time” in deciding to become circumcised and in moving forward with their decisions.

### Pre-intention Stage Awareness

#### Exposure to VMMC messages

VMMC-related behavior change begins when the idea of adult male circumcision enters a man's consciousness. We identified 34 unique sources of VMMC information in the interviews and men cited on average four different sources (Table 2.A). Overall, personal network members were the most important source of information cited by the men. Over time, repeated exposures to VMMC messages coming from various corners of men's lives amass into a “social proof” [29] that VMMC is valuable. When this occurs, men begin to pay attention:

**Table 2:** MMC Information Sources, Perceived Benefits, Critical Belief Adjustments, and Major Fears Cited by Ever- and Never-Married Status (n = 40).

	Cited		
	Ever-Married (n = 17)	Never-Married (n = 23)	Total
**A. Source of Information**			
Social network member			
• Male friend, school- or work-mate	13	22	35 (88%)
• Wife or girlfriend	14	9	23 (58%)
• Family member	7	14	21 (53%)
• Faith leader	1	1	2 (5%)
Media			
• TV or radio	9	8	17 (43%)
• Print media	8	7	15 (38%)
• Internet	1	2	3 (8%)
Health program or campaign	4	7	11 (28%)
Health provider or counselor	7	3	10 (25%)
Average number of different sources	3.7	4.2	4.0
**B. Perceived Benefits**			
HIV/STI prevention	15	22	37 (93%)
Hygiene, being clean	13	16	29 (73%)
Prevents cervical cancer	7	12	19 (48%)
Female pleasure/male sexual performance	9	9	18 (45%)
Prevents cracks, bruises, and abrasions	5	3	8 (20%)
**C. Critical Belief Adjustments**			
MMC is for Christians too	3	7	10 (25%)
MMC is safe	6	3	9 (23%)
MMC is appropriate for adults	6	1	7 (18%)
MMC is appropriate for my ethnic group	5	1	6 (15%)
**D. Major Fears**			
Wound care and healing	8	16	24 (60%)
Pain and injections	8	12	20 (50%)
Adverse events and outcomes	7	6	13 (33%)
Service issues	4	5	8 (23%)
Other concerns	2	8	10 (25%)

*I kept seeing these guys going for circumcision and they were telling me, ‘It keeps a man clean.’ But I doubted them and was saying, ‘If it's so important why didn't God think to remove it before creating us?’ I didn't see the importance. Then a friend went for it, so I asked him why. I wanted to find out the truth about it and how they do it.... Early this year is when I really started to think about it. It was widely publicized, and I was like, ‘Wow, this thing is serious.’ What can I say? I was just, like, overwhelmed* (21 years old, never-married).

Hearing about VMMC from multiple sources is clearly important, but learning about it from “guys like me” is especially influential. Almost all of the men described encounters with friends, school-, and work-mates during which they began to formulate an understanding of VMMC and how it confers health and hygiene benefits (Table 2.B):

*My friends explained that if you're not circumcised dirt gathers in the foreskin and if you go for several days without bathing you can infect your wife with cervical cancer. If you're circumcised, then diseases like syphilis also don't have a place to stay, they just dry off* (32 years old, married).

The notion that the foreskin harbors “dirt” (*doti*), moisture and disease recurred frequently in the interviews, rationalizing its removal to keep the penis *open*, *clean*, and *dry* and to avoid giving diseases *a place to hide*. Describing their experiences with *cracks*, *abrasions* and *bruises*, which they believed resulted from movement of the foreskin during sex, eight of the men further described a morphological advantage for foreskin removal. Reducing the *high temperature* during sex, perceived to cause premature ejaculation, almost half of the men mentioned prolonged erection and, consequently, enhanced sexual pleasure for women as another morphological advantage of circumcision. When such perceived advantages are grounded in the private details of a friend's actual sexual health and circumcision experience, explanations of VMMC benefits become even more credible:

*My friend, he's married too, he told me that he used to have this white stuff, then cracks and an itch, just like me. Then he got circumcised and it all stopped* (34 years old, married).

*I proposed to this girl and she refused saying only circumcised men are good in bed. So I asked my friend who is circumcised and he agreed, telling me*, ‘My wife, she doesn't give me a break anymore. She wants sex everyday now’ (23 years old, married).

Even at this early stage of change, men are also curious about VMMC surgery and many are eager to learn *the truth* about how it is done. Able to demystify the procedure and to share relevant information that other men can relate to, close friends who can tell-the-tale about what they personally went through are particularly convincing:

*I started to see it wasn't dangerous because my best friend from the Copperbelt did it. He even showed me. I then decided to talk to my wife about it* (28 years old, married).

The role of male friends and peers as catalysts to get other men to take VMMC seriously did not appear to differ substantially between ever- and never-married men.

This was not the case as relates to female partners and other family members. Including mothers, fathers, aunts, uncles, brothers, sisters, cousins and grandparents; younger, never-married men cited more family members compared to older men (Table 2.A). Although influential – e.g., *My father sat me down and we had a man-to-man talk* and *My mom pushed me to go* – family members' influence was generally limited to raising the topic of VMMC and encouraging the young men to consider it. Current and future female partners, on the other hand, wielded quite a lot more influence over the men and in more diverse ways. Women's influence was most important in the later stages of behavior change, but the example of “Alice” demonstrates the potentially critical role that female partners can play in introducing VMMC to men:

*I met a girl named Alice* [who later became his fiancée]. *She's the one who enticed me to do it. She came with a brochure and said*, ‘You know honey, there's something we can do to make our love more enjoyable.’ *She will be my first woman and I will have children with her. Whatever she tells me I believe out of love* (27 years old, never married).

#### Critical belief adjustment

Twenty of the 40 men described specific changes in their beliefs about circumcision that opened up the possibility for them to contemplate the procedure (Table 2.C). These critical belief adjustments concerned the safety of VMMC and its appropriateness for men like themselves:

As Christians, *Jesus was circumcised, so I thought even me, as a Christian, I need to lead a Christ-like life* (18 years old, never-married).

As older men, *People were saying MC is for the younger ones and that I'd be lowering myself if I went for it. But this MC program shows us how to keep ourselves as men* (40 years old, widowed).

As men coming from a non-circumcising ethnic group, *It used to be only for North Westerners because it's in their tradition. Then the government proved it reduced sexually transmitted diseases* (48 years old, married).

The older, ever-married men in our sample cited more critical belief adjustments than the younger, never-married men (Table 2.C), a finding worth investigating in future research in representative samples.

### Intention Stage Decision-Making

#### Social pressure

In the intention stage of behavior change men decide that they want to be circumcised. In 26 of the interviews men described explicit social pressure to become circumcised that pushed them to decide to go for it themselves. One form of social pressure derived from individuals' perceptions that circumcision was the new norm:

*This is an honest opinion, everyone around me has gone for it so I feel different. I was being neglected. Now I just feel like I'm doing what a man is supposed to do* (21 years old, never married).

Whereas men like the one quoted above expressed internal anxiety about being different from a vaguely specified *everybody else*, other men described more direct pressures exerted by their male friends:

*When we're bathing my friends ask me why my penis looks like a pen. They tell me, ‘Go and remove that foreskin!’ I admired them because they'd done it. I wanted to be smart too* (33 years old, married).

Pointed pressure from one individual, especially a good friend, was also highly influential:

*I didn't give it much thought at first but then my friend decided to get circumcised and he challenged me to do it too. That was about three months ago. Then he went for it!, and I started to think it's high time that I do it too* (26 years old, never-married).

Whether from the self perception of being different from others or from the experience of direct pressure from others to become circumcised, many of these 26 men expressed a sensation of missing out and being left behind:

*I started to feel like I was missing out on a really big thing. Like when you forget your phone, you feel like something's missing and then you realize*, ‘Oh, I left my phone’ (25 years old, married).

Virtually all of the men in relationships with women reported varying degrees of persuasion-to-pressure from their partners, although relatively few described this as being forceful or insistent. Two men, however, reported that their fiancées made circumcision a pre-condition to marriage, while another explained that his girlfriend emphatically demanded that he get circumcised (*You know how women are, she's the reason I'm here today*) as a way of resolving her recurrent fungal infection. Our data do not show substantial differences in the experience of social pressure between older and younger, ever- and never-married men in our sample. However, we did not probe age-specific issues that may have revealed ways in which these two categories of men differentially perceive and respond to social pressures they experienced to become circumcised.

#### Personalization of VMMC benefits

Personalization of VMMC advantages was the most consistent yet diverse behavior change trigger observed in the interviews. By personalization we refer to a process wherein men reformulate and fix abstract concepts of VMMC benefits – e.g., reduced sexual risk – to their precise individual needs. Rather than “perceived advantages,” we coded to the personalization category men's specific personal reasons, rationalizations, and justifications to go ahead with an otherwise unpleasant and inconvenient surgical procedure. All but one man described one or more specific personal reason for becoming circumcised, the majority relating to perceived sexual risk, which was communicated as:

A distrust of himself: *We drivers move from here to there and I may accidently meet a beautiful girl and, you know, I don't know how she is* [referring to her HIV status]. *Maybe I'd be drinking and have sex with her without a condom. Without that preventive measure, I could wake up the next morning and it's too late. So I could've shortened my life and even my children's too* (48 years old, married).

A distrust of his partner: *I can take care of myself, but I don't know what my wife is doing* (26 years old, married).

An ostensible concern for his partner's wellbeing: *I was concerned about my wife, that I might infect her with diseases, so I decided to get circumcised to be free from any worries* (32 years old, married).

A partner's concern for her own wellbeing: *When I have sex with a woman I get these cracks. So I talked to my wife about circumcision and she said*, ‘You men are difficult. Just go.’ *I then went to X clinic but I couldn't go through with it. I turned around at the gate* (23 years old, married).

Motivations related to hygiene – being *clean* and *smart* – were similarly grounded in and rationalized by the individual contexts of men's lives, and ranged from concrete needs – e.g., *getting rid of bad urine smells* – to more encompassing concerns – e.g., *getting clean before for the marriage thing*. As with sexual risk reduction, the hygiene advantage of VMMC had particular appeal to men who were often on the road:

*Like me, I travel a lot and the places I go may not have water, so the penis gets dirty. Looking at this work I do, I thought when I get circumcised even if I go for two days without bathing, I'll be clean. A man needs to be smart in his life* (33 years old, married).

Triggered by their partners' request or in anticipation of a future relationship, circumcision in preparation for marriage was cited frequently by unmarried men. Alluding to the possibility of remarrying someday, one widowed man explained:

*When I heard that MC prevents cervical cancer, this touched me a lot because my late wife died of cancer. It was a tumor on the neck. This made me feel guilty that maybe it happened because I wasn't circumcised* (40 years old, widowed).

The example above is interesting on two counts. In linking his decision to become circumcised to his late wife's fatal illness, it illustrates well the great specificity of personalized VMMC advantages. In apparently confounding cervical cancer and neck tumor it also demonstrates the significant degree to which some men reinterpret public health messages to produce new and particular understandings of VMMC, thereby rationalizing its unique value for their own lives.

The manner in which four men reinterpreted the relationship between VMMC and STIs is worth noting. Describing the experience of STI symptoms, apparently from persistent infections, two men decided to go for VMMC as a way to resolve the recurrent problem while two other men wanted to prevent new STI infections in the future:

*I had ‘licking,’ a disease when you have pus that comes out of your penis, and syphilis. My friends told me to first get medicine and then go for MC when I'm healed so I'd be protected from these diseases. The people at X clinic gave me details and I realized that if I had been circumcised I wouldn't have gone through what I did* (31 years old, married).

None of the four men mentioned condom use or sexual partner reduction as a means for preventing STIs.

The very nature of the personalization theme defied easy categorization of the men's replies. Nonetheless, being *clean*, being *safe* from disease and being *sexually appealing* were predominant and over-riding motivations.

#### Fear management

All of the men cited one of more fears about the surgery and the healing period (Table 2.D), but 32 individuals described going through variably long attempts to overcome their fears about getting the surgery. The significant degree to which some men struggle with fear is striking. Claiming to have gone through a 10 year period between the time he decided to become circumcised and the date we interviewed him, one participant explained:

*Your heart is willing, but you have these fears. It's only the fear that holds us back. It took me a long time to come here, 10 years, that's a long time. But now my mind is set and I am free* (33 years old, married).

Whether or not “10 years” accurately reflects the time it took this man to make his way to the VMMC clinic, his experience of being “held back” by fear represents the experience of many other men in the sample.

Getting their specific questions answered – how the surgery is done, the risks involved, how to care for the wound and how long it will take to heal – is critical at this stage. Although several of the men expressed an interest in learning about procedure-related issues from health professionals, few indicated that they had tried to get information about VMMC by visiting a clinic. Most men who went to VMMC clinics to learn more about the procedure left unsatisfied. This dissatisfaction was mainly due to long queues (e.g., *The crowds were too big so there was no time for counseling*, 25 years old never married), to incomplete counseling (e.g.,. *All they talked about was the six week period of abstinence, whether you're married or not, and I didn't get all my questions answered*, 34 years old, married), and to inconsistencies in information received:

*Even though there was a poster advertising MC, when I asked who could help me the first person told me* ‘I don't know, ask somebody else.’ *The next person I asked told me they offer it everyday but somebody else said they did it only on certain days. I didn't have a clear picture and so ended up asking my brother about the cost and my friends explained about the follow up visits* (26 years old, married).

Asking health provider friends or family members for information or going to private clinics produced more positive experiences. Covered by his employer's health plan, this individual explained: *I had a lot of questions so I went to a clinic in my health scheme. I got most of my questions answered. Then it reached a point where, like, everybody was doing it and I just had to do it too* (34 years old, married). Having had his many specific questions answered clearly readied this man to move forward when the time was right.

Given the difficulty in accessing information from health professionals, men relied heavily on their friends, not only for information but also for moral support to build up the courage to proceed:

*Last year I made up my mind to do it, but I had this major concern about the recovery and the pain. I started out to a clinic twice and both times changed my mind. It just takes counseling. When you're afraid of something, you need to be counseled. Most of that came from my friends. They encouraged me, saying*, ‘Come on, I've gone through it. You can do it too.’ *That is how I managed to get over my fears* (20 years old, never married).

As men approach a definitive commitment to go for circumcision, they also turn to their partners for support. Thirteen of the 17 married men and eight of the never-married men specifically asked their female partners to give their consent for them to go ahead with the surgery. At the same time, allowing them to muster enough courage to just go and *get the job done*, up to the time they present at the clinic men describe a kind of self-counseling to remind themselves that *no pain lasts forever*, *all wounds eventually heal*, and *a lot of other guys have done it and are walking around*.

### Action Stage VMMC-seeking

Having struggled for months or even years, by the time men manage to contain and set aside their fears some are quite eager to be circumcised:

*I'm ready now, I even feel desperate. It's like having a thirst. I need to drink water* (33 years old, married).

Thus committed to going through with it, the challenge then becomes finding the time. Fitting VMMC surgery into their schedules was most challenging for working men as well as for men who traveled frequently. Trying to fit VMMC surgery in over a weekend or during a short break, men often had only a few days or a week to undergo and heal from the surgery before having to report back to work. Constrained by his security guard work, one individual had to squeeze the surgery in between two nighttime shifts: *I knocked off this morning and came straight here. I just told my wife I was going to the clinic. I'll go back to work this evening* (35 years old, married).

Beyond thinking that they might be able to fit the surgery in over a weekend or during a break from school or work, very little additional planning was described by the 40 men. In reply to our questions about the preparations men took to make it to the clinic that day, close to half of them said they had decided that morning, e.g., *Today I just woke up and had the spirit. I woke up and said,* Today, I'm going to do it (19 years old, never married). This *today-is-the-day* planning style surely contributed to failed prior attempts to become circumcised.

Unprompted, 17 of the men mentioned that they had unsuccessfully sought VMMC surgery one or more times in the past. Of these individuals, 12 were on their second attempt, four their third attempt, and one man was on his fourth attempt. All told, we identified 22 incidents of failed prior VMMC attempts in the interviews. These included 13 incidents of service unavailability stemming from men presenting on the wrong day or arriving late, clinic over-crowding and being turned away, and service cancellations. Eight other unsuccessful attempts were due to unacceptable service conditions, including a lack of privacy, female providers and concerns about the quality of the clinic. One case was not clear from the interview.

Appreciating the complex interaction between demand- and service-side barriers is important for understanding missed opportunities for VMMC delivery. The example of a 34 year old, married man who took several years to determine that VMMC was right for him and to overcome his fears illustrates:

*Okay, to tell you the truth, about 95% of my fears are gone. A while ago I actually went to X clinic during the week, but was told they only do it on Fridays. I went back that Friday and had to make a u-turn. I had this fear that they wouldn't do it well. Later I decided I should have it done at Y clinic instead. Now I'm on leave from work so I wanted to get this done. Anything can come up so I thought I better do it this week to allow me to heal before I have to report back at work. This morning I decided*, ‘Whether or not I still have any fears, today I just have to do it.’ *So I went to Y clinic but found it was closed because a celebration of cancer day. I went home and just sat my chair, thinking. Since I decided that I should do this today, I thought I'd try this clinic because they can do it well* (34 years old, married).

As it turned out, the day we interviewed this man there were too many clients so he ended up being turned away and asked to return the next day. As this individual was clearly motivated and seemed to have the time, it is likely that he went back and was served. Other men might be less inclined to return:

*I gave this a lot of thought yesterday and knew that I had to do this today. I knew if I had any procrastination today I wouldn't go through with it and it would take another year. I decided, ‘Let me just go,’ so I told my supervisor that I had a personal errand and then I stormed out* (34 years old, married).

As we did not explicitly ask about prior VMMC-seeking attempts, the number of unsuccessful VMMC attempts we found in our data may be under-representated.

## Discussion

The present study advances our understanding of VMMC uptake from both demand- and supply-side perspectives. By grasping demand as a behavior change process, rather than as a static, either-or state, our findings highlight opportunities to accelerate men's movement through the process of change. From the men's VMMC-seeking accounts our study also identifies the ways in which service delivery weaknesses obstruct and delay surgery uptake. To achieve ambitious VMMC scale-up targets in Zambia and elsewhere, capitalizing on demand-side opportunities while addressing the supply-side issues will both be needed.

Two broad opportunities to hasten the behavior change process are evident in our data, one relating to the messages and the other to the messengers. As relates to the former, our findings clarify the need to tailor messaging to men's stage-specific needs and concerns. Bearing out a health promotion truism, our findings on the pre-intention stage indicate that men sat up and took note of VMMC when they heard multiple messages from a variety of different communication channels. The key communication goal at this stage should be to provoke men to rapidly shift from having a general awareness of VMMC to reflecting on the possibility of circumcision for them selves. Widely cast awareness and education campaigns combined with more targeted messaging for older men, Christians, and men from non-circumcising ethnic groups to speak to their hesitations would be effective.

Once men start to entertain the possibility of VMMC for themselves, the communication focus needs to shift to intention stage priorities. Programming for this stage should push men from merely contemplating the possibility of VMMC to making a decision that they want to be circumcised. Evidence from our sample suggests that this is occurring “naturally” due to increased social pressures to become circumcised. At the same time, our findings demonstrate how men ascribe particular value and meaning to circumcision to fit within the unique contexts of their lives. From a social marketing perspective, therefore, appealing to men's individuality and to their sense of self as *clean*, *free*, *sexually appealing*, *responsible*, *in control*, and *safe* men, could hasten the VMMC decision-making step. Yet, caution is required in developing social marketing messaging strategies. Although not the intent of the study, our findings on the way men rationalize VMMC – e.g., to be *clean*, *safe*, and *free* of STIs – demonstrate an exaggerated sense of protection and hence the potential for risk compensation [30].

Perhaps the greatest opportunity to increase VMMC uptake that came out of our findings pertains to shortening the duration between men's intentions to become circumcised and their actual VMMC-seeking. Helping men to overcome their fears about the procedure is critical in this regard. Men need to be informed about the procedure and how it is done. They also require specific information about what they need to do to prepare for the surgery and to ensure proper healing. While some procedure-specific information can be provided through broad-based educational campaigns, men have different questions and concerns and queuing up at crowded heath facilities to get their specific questions answered is not a viable option. Alternative approaches should be tried to increase access to information from health professionals. VMMC information booths that are strategically placed at workplaces and public locations, and which could accommodate one-on-one counseling would be ideal. Introducing or expanding VMMC telephone help-lines may be a partial solution for individuals who have little free time or who are frequently on the road.

Given the particular influence of male friends and female partners at all stages of the behavior change process, interventions to reach men via friends and partners are especially promising. Frequent references to work- and schoolmates in the interviews suggest that peer education programs in institutional settings could be an effective intervention and such programs should be implemented or intensified where VMMC uptake remains low. Our data also suggest that conventional peer-to-peer approaches should take better advantage of the uniquely influential role of close friends by tapping directly into friend-to-friend encounters, in particular those that involve VMMC-experienced men. Engaging and deploying post-procedure clients as “VMMC buddies,” tasked to educate and support their non-circumcised friends to move forward with the surgery, is one possibility worth exploring. The potential to influence men via their female partners also came out clearly in the study. While stepping up efforts to reach uncircumcised men through their personal networks of male friends, programs targeting women in church, community, and clinic settings, and equipping them to effectively promote VMMC to their partners, should also be fortified or developed. Such a strategy could prove especially effective to increase uptake among older men.

The importance of minimizing supply-side failures to deliver VMMC to men who are ready can be more fully appreciated with our improved understanding of what men go through before presenting at VMMC clinics for the surgery. Years may pass in the time it takes a man to become aware of VMMC, decide that it is right for him, to muster the courage to undergo the surgery, and to find the time to go to the clinic. To be turned away – as many men in our sample were – may not only delay the performance of circumcisions, it could discourage some men from returning to the clinic.

As noted above, men have difficulty accessing credible *and* accurate information about VMMC at all stages of change. That information was difficult to access was a function of both the location of the information (generally only within health facilities) and the limited number of sites. Telephone help-lines, workplace education programs and drop-in counseling centers in business and trade hubs could help reduce information access barriers. Limited physical access to services is a greater challenge. Expanding the number of VMMC sites to address this issue would be the most obvious solution but remains dependent on a range of health system inputs, most notably an increase in trained personnel. Improving clinic efficiencies in processing clients as well as introducing time-saving procedures, e.g., using VMMC devices instead of surgery, is perhaps more realistic. Additionally, advance-scheduling options should also be considered to help address uniquely challenging barriers to service access experienced by working and traveling men.

The men in our sample also indicated that the clinic environment, encompassing both physical and relational spaces, was a supply side weakness. Insufficient space allocated to VMMC services within the clinic led to overcrowding and a lack of privacy. Some men described how these physical conditions were exacerbated by the weak interpersonal relationship with healthcare providers who lacked the time to deliver adequate pre- and post-procedure counseling or to answer their questions. Such in-clinic conditions both increased men's doubts and fears and made the decision to follow through with surgery that much more difficult.

The supply side problems identified in this study are reflective of broader health system weaknesses, rather than VMMC-specific issues. Yet our findings reconfirm the way demand and supply for any health service co-exist and influence each other. To date, more emphasis has been placed on researching and developing strategies to promote and increase demand to meet ambitious scale-up targets for Zambia. Meeting the demand for VMMC in the current service delivery conditions, however, seems unlikely.

### Study Limitations

However revealing our findings we recognize some important limitations in the study. First, interviewing adolescents waiting to be circumcised would have improved our understanding of the differences in motivations, barriers and decision-making contexts for younger and older men. Second, not asking men explicitly about unsuccessful prior VMMC-seeking and their motivations to keep trying to obtain the procedure is a limitation in our data. We recommend that future qualitative and quantitative studies include questions to explore these topics in more depth. Finally, while the small sample and in-depth interviews were appropriate to the purpose of our study, follow-on research in large populations of men would add valuable insight on the duration and nature of the behavior change process leading to VMMC uptake. A greater understanding of VMMC-seeking barriers among men who have yet to commit to having the procedure done should also be prioritized in future research. We finally acknowledge that conducting the interviews immediately prior to the scheduled operation may have affected (positively or negatively) the men's decision to go through with the procedure. Future studies should consider interviewing post-operative men instead of individuals waiting for the procedure.

## Supporting Information

Table S1 **Fifty-Five Initial Themes Derived From** ***In Vivo*** **Coding with Exemplary Quotes.**
(DOCX)

Materials S1 **Interview guide.**
(PDF)
