# Correction: Behavior Change Pathways to Voluntary Medical Male Circumcision: Narrative Interviews with Circumcision Clients in Zambia

**DOI:** 10.1371/journal.pone.0116361

**Published:** 2014-12-18

**Authors:** The PLOS ONE Staff

There is an error in the third affiliation for the fifth author, Stephanie M. Topp. The correct affiliation #5 is: University of Melbourne, Nossal Institute for Global Health, Melbourne, VIC, Australia.
