# Supplementary material for: Behavior Change Pathways to Voluntary Medical Male Circumcision: Narrative Interviews with Circumcision Clients in Zambia
Source: PLoS One. 2014 Nov 6;9(11):e111602. doi: 10.1371/journal.pone.0111602 (PMC4222873; doi:10.1371/journal.pone.0111602)
Supplement: Table S1 — Fifty-Five Initial Themes Derived From In Vivo Coding with Exemplary Quotes. (DOCX) [file pone.0111602.s001.docx]

**Table 1: Fifty-Five Initial Themes Derived From *In Vivo* Coding with Exemplary Quotes**

| Theme | Exemplary Quote |
| --- | --- |
| Influence from: |  |
| 1. Female partner | *First I heard about it on TV. After that I had unprotected sex I developed some stuff like wax. I was told that the foreskin gets germs, for what reason I don’t know. After getting this wax me and my girlfriend decided to at least come for MC.* |
| 1. Father | *My father just told me and my cousins, ‘You guys should go through the male circumcision.’ My cousins did it last month. I was planning to undergo it next year but I just changed my mind and decided that I should just do it this year.* |
| 1. Mother | *A friend of mine on campus talked to me about the benefits. I’ve seen it advertised a lot on TV, ‘A man who cares . . .,’the ads like that. I read about it online too. The benefits and reducing the risk of STIs, that’s what triggered me. Then my mom, she’s a nurse and works at the Ministry of Health, she talked to me about it and that’s encouraged me too.* |
| 1. Brother, male cousin | *I starting hearing about MC a long time ago but I never thought I’d come for it. But after my brothers and my friends gave me ideas, that’s when I realized it’s serious because people can’t just say things without knowing.* |
| 1. Other relative | *My uncle was also advising me and asking me, ‘Have you been circumcised?’ When I said ‘no’ he told me, 'You just have to go do it, it helps.'* |
| 1. Male friend | See codes #3 & 4 |
| 1. Co-worker, school-mate | *We were bathing at my work and I noticed my workmates were circumcised. I asked how they did it. Then said we just did it recently and told me if I wanted I could also get circumcised. There are a lot of clinics I could go to.* |
| 1. Health worker | *I heard about it at from a workmate and the doctor where I work. She gave me a phone number and fliers.* |
| 1. Counselor | *I also talked to a marriage counselor and another elder, the ones you call bashibukombe. I was just getting views.* |
| 1. Religious figure | *My pastor said I should also go. He said even Jesus was circumcised.* |
| 1. Peer educator | *There is this peer educator who told us about the good part of male circumcision. So it was more like there was a workshop in Chilenje and one of the topics was about STDs and HIV/AIDS prevention.* |
| 1. Mass-media (TV, radio) | See code #1 |
| 1. Print media | *I knew of this place a long time ago. I used to pass by and would see the poster [for VMMC].* |
| 1. Campaign, outreach | *It was first at school at a boys networking club. They called all the boys into the assembly and talked about male circumcision.* |
| 1. Other social influence | *I am almost in the process of getting married, so I want to be clean before the marriage thing, that’s is the other thing that triggered this circumcision process.* |
| Perceived advantages: |  |
| 1. Clean, hygienic | *How can people be talking all the time about something that is not very special? All the time someone is encouraging you, saying it’s a good thing because you won’t have cracks on your penis or white stuff, that is talk of hygiene about white stuff normally found on the penis. So I thought like me I think let me try to do the same.* |
| 1. Appealing | *What happened was I was chatting with a friend at work he was saying that if we were both going after the same lady she’d never go out with me but would go with him because he is circumcised.* |
| 1. Sexual performance | *For me I think what triggered it that time is that somebody told me that it also helps to minimize the problem of premature ejaculation. I had that problem and I was advised that once you cut off the foreskin it helps. That's what immediately triggered it.* |
| 1. Cracks, bruises, cuts | See code #18 |
| 1. HIV/AIDS | *What I’ve heard is that when a guy is circumcised, he will not be getting sick with diseases, but only big ones like HIV/AIDS and other STDs, like syphilis.* |
| 1. (Cervical) cancer | *With the foreskin is cut it helps you in protecting against STDs and other diseases. I heard if you are not circumcised and having sex with a woman you can make her sick, she can get cancer.* |
| 1. Reduces risk of STDs | *There was one who was circumcised and he is the one who was saying that if you get circumcised you will reduce the risk of getting sick from gonorrhea and syphilis.* |
| 1. Specific problem resolution | *The doctor told me that they would give me medicine for this problem of warts and that I needed to go for male circumcision.* |
| Fears/barriers: |  |
| 1. Pain | *I was thinking that maybe I'll just induce pain on myself, so why should I bother.* |
| 1. Procedural error | *At first I thought they may cut the veins and then maybe I’d stop functioning properly.* |
| 1. Bleeding | *I started thinking about it in September. It was the season. Some guys said you don’t go for circumcision in the hot season because you’ll bleed a lot and it will be like an injury.* |
| 1. Wound healing | *My concerns about having it done were that maybe if the wound won’t heal quickly what will I do? What if the bleeding continues, what will I do? And then the other issue, like I said earlier, what if they cut my skin and it doesn’t go back to its normal shape. I was also worried about that.* |
| 1. Healing time | *There is this guy who underwent circumcision and it really took long to heal, so that made me afraid.* |
| 1. Becoming impotent (“lame”) | See code #25 |
| 1. Loss of sexual sensation | *My first impression was that, obviously, it was a very painful procedure. Also, I heard that it could take away some sensation when having sex, like some sexual pleasure.* |
| 1. Injection, stitches | *The stitches. I have never gone through the surgery process so I don’t know about the stitches.* |
| 1. Female provider | *When I first went I was told that a woman is the one going to be doing the surgery. This was a major worry. Another woman [other than wife] touching my balls?, this was a major issue. You know what usually happens when a woman touches your balls, things will get sour. I will end up exploding like Hiroshima or Nagasaki. That is the worry that I had. I talked about it with my wife and we decided I shouldn’t to go there.* |
| 1. Provider competence | *The major concern was that since they are using these machines, technology they sometimes just get tired and maybe the doctor working on you just may make a mistake, maybe cut something.* |
| 1. Privacy | *My concern is, like, us men, we like taking this as a private, confidential kind of thing that no one else other than friends are supposed to know. So my major concern is how I will be able to conceal it.* |
| 1. Obligations, time | *I was doing water engineering, which involves a lot of physical work, but less paper. I heard it takes a week for one to get back to working so I thought maybe a week would be too much time for me.* |
| 1. Transportation, access | *I’m coming from a very far place and I don’t know how much pain I’ll feel because I’ll be going on foot and the place is very far.* |
| 1. Fees | *I also use to think on circumcision but was wondering if they charge for it. Then I decided that I should just come and learn about it from here.* |
| 1. Other | *When you’re cut, your walk will change, that’s what was in my mind.* |
| Changes in beliefs, attitudes: |  |
| 1. Adult men | *I felt very encouraged [after learning about MC] because most of us Zambians are from tribes that aren’t circumcised, but I started to feel this thing [MC] is good. Even if I’m older, this thing is good. So it came to my mind is that I have to be one of them.* |
| 1. Ethnic group | See code #39 |
| 1. Religion | *Even in the bible, it’s written you need to be circumcised.* |
| 1. Safety of procedure | *I saw that there are so many people that have been circumcised and they are living, walking around, so who am I not to join the team or to be one of them. That also encouraged me.* |
| Self-perception: |  |
| 1. Responsible | *I’m engaged [to be married] and . . . I just want to be responsible.* |
| 1. Free, empowered, safe | See code #47 |
| 1. Desirable | *What I came to know is that when you are circumcised, ladies enjoy sex.* |
| 1. Clean | *By that time I was thinking I just have to be gentle, I just have to be a clean man* |
| 1. In control, in charge | *If I do this thing, I’ll make progress in life. (In what way?) I’ll have already prevented diseases, mostly the sexual diseases. Like, I’m a driver, we move here and there. What happens in Livingstone is not what happens in Nakonde. Maybe today I will be in Livingstone and the other day I will be in Mwinilunga. You never know. I’m a man and I have feelings. It’s better I go for preventive measures so that I will on the safe side like others.* |
| 1. Belonging | *More especially from where I’m from almost all of my neighbors have done it. I never want to be left out.* |
| Social pressure: |  |
| 1. Pressure from friends | *One thing that made me like come to a decision to do it is because my friends have done it. I’m the only one who hasn’t gone. So, yeah, I was feeling uncomfortable when my friends are talking and teasing me that I’m not clean and things like that.* |
| 1. Pressure from female partner | *My girlfriend, I’ve actually been getting some pressures from her, like ‘Just do it.’ She’s been pressuring me to do it though we don't do sex. She says it’s good for my life.* |
| 1. New norm | *It reached a point where like everybody is doing it and I had to do it too.* |
| Fear management: |  |
| 1. Self-counsel | *As a matter of fact people heal. So I just looked at it and said I'll heal one day and it will be soon. So like for bleeding, I’m sure it won't even take a whole day. I just told myself that it’s a process that I have to undergo. It won't last forever.* |
| 1. Forget, ignore | *I just stopped thinking about all the stories that I heard, like that the shape [of my penis] may change or that maybe I’d have prolonged bleeding and stuff like that. I just stopped thinking about these things and only wished to come for circumcision.* |
| 1. Committed, eager | *When you trigger your mind saying, ‘I need it and I need it now,’ that kind of passion in something can let you do anything. You know, you give up concerns and everything.* |
| 1. Others manage, normal healing | *The fact is that I know that one day it will be healed and I will be just like any other guys who have done it. If others have done it I can also do it too.* |
