# Supplementary material for: Behavior Change Pathways to Voluntary Medical Male Circumcision: Narrative Interviews with Circumcision Clients in Zambia
Source: PLoS One. 2014 Nov 6;9(11):e111602. doi: 10.1371/journal.pone.0111602 (PMC4222873; doi:10.1371/journal.pone.0111602)
Supplement: Materials S1 — Interview guide. (PDF) [file pone.0111602.s002.pdf]

Evaluation of Acceptability and Demand for VMCC and the Prepex MC Device

11/08/12 Version: Protocol

Behavior Change Narrative Interview Guide with MC Clients

Respondent ID: ☐☐☐ Date: ☐☐ / ☐☐ / ☐☐☐☐ (d d / m m / y y y y)

Interviewer ID: ☐☐☐ District:

Clinic ID: ☐☐☐ Recording ID: ☐☐☐

Section I: Background

Question No	Question	Coding Categories	Skip to:
**101**	How old are you?	______ [write in years]	
**102**	[[Have you attended school?]]	yes ☐ 1<br>no ☐ 0	 → Q104
**103**	How many years of school have you completed?	______ [write in years]	
**104**	What is your current marital status?	never married ☐ 1<br>divorced ☐ 2<br>married / cohabitating ☐ 3<br>widowed ☐ 4	
**105**	What is your religion?	Christian ☐ 1<br>Muslem ☐ 2<br>Hindu ☐ 3<br>None ☐ 4<br>Other ☐ 5	
**106**	What tribe are you from?	Tonga ☐ 1<br>Bemba ☐ 2<br>Nyanja ☐ 3<br>Lozi ☐ 4<br>Lenge ☐ 5<br>Luvali ☐ 6<br>Chewa ☐ 7<br>Kaonde ☐ 8<br>Other ☐ 9	
107	When did you first hear about male circumcision for adult men?	year____month ____	

Thank you for giving me this information. As I explained before we started the interview, we are interested in understanding how you learned about male circumcision and how and why you came to decide to get circumcised yourself. I'm going to ask you a few broad questions about your personal experience. Tell me as much detail as you wish and please emphasize aspects of your experience that were particulary important for you in the process of deciding to have the procedure today.

Section II: Behavior Change Process		
**Question Code**	**Question**	**Sub-codes**
**Step 0**	1 What did you hear about male circumcision for adult men?<br><br>2 How? Where ? From whom?	first exposure<br><br>exposure context
**Step 1**	3 Describe your first impressions and thoughts about it?<br><br>4 What did you learn about it?<br>Probe: Advantages for men in being cicumcised? Health benefits? Information about the procedure?<br><br>5 When you first learned about MC, did you think it was relevant to you personnally? Why? Why not?	first impressions<br><br>initial information<br><br>personal relevance
**Step 2**	6 When did you start thinking about the possibility of male circumcision for yourself? In what context?<br><br>7 What triggered you to start thinking about it for yourself? Did anything change? Did some one you know personally talk to you about it?<br>Probe: A friend had one? Your partner mentioned it?<br><br>8 At this time, when you were contemplating the possibility of becoming circumcised yourself, what were your concerns about having it done? What were your major	early contemplation<br><br>early triggers<br>positive social support<br><br>early fears
**Step 3**	9 Despite your concerns about MC, what made you start thinking about it seriously for yourself?<br><br>10 Did you talk to anyone at this point about your contemplation of getting circumcised. Who? (Probe: Friend? Partner? Provider?) How did they influence you in making the decision to get circumcised?<br><br>11 Where did you go to get more information on the MC procedure? Did you have any trouble finding a place to get information?<br><br>Were all of your questions answered? (Probe: about the procedure, recovery time, cost, risks, where to get it done?)<br><br>12 By this time, had you overcome your major concerns and fears about getting circumcised? Why, what happened?<br><br>13 Did anything else continue to concern you about getting circumcised? What? Did you do anything or talk to anyone at this point to help you resolve these concerns?	early action<br><br>positive social support<br><br>information seeking<br>information access<br><br>attenuated fears<br><br>continued fears<br>fear resolution

Question Code	Question	Sub-codes
**Step 4**	14 So you decided that you wanted to get circumcised. Describe to me everything that you had to do to make today's appointment. (Probe: Physically come to clinic? Take time off work?)<br><br>How did you know about this clinic?	planning steps
	15 In order to go through with this procedure today, what did you have to arrange at work? At home?	preparations
	What part of preparing for the surgery did you find most challenging? How did you overcome these challenges?	planning challenges/barriers
	16 Now that you are ready to have the procedure, do you continue to have any concerns about it? What are they? Despite having some concerns, what makes you want to go ahead with the circumcision?	persistent fears<br>fear resolution
